# Blood pressure, hypertension and the risk of abdominal aortic aneurysms: a systematic review and meta-analysis of cohort studies

**DOI:** 10.1007/s10654-019-00510-9

**Published:** 2019-03-22

**Authors:** Elsa Kobeissi, Makoto Hibino, Han Pan, Dagfinn Aune

**Affiliations:** 1 Department of Epidemiology and Biostatistics, School of Public Health, Imperial College London, St. Mary’s Campus, Norfolk Place, Paddington, London, W2 1PG UK; 2 Department of Nutrition, Bjørknes University College, Oslo, Norway; 3 Department of Endocrinology, Morbid Obesity and Preventive Medicine, Oslo University Hospital, Oslo, Norway

**Keywords:** Hypertension, Blood pressure, Abdominal aortic aneurysm, Systematic review, Meta-analysis, Cohort

## Abstract

Abdominal aortic aneurysms (AAA) are fatal in 80% of the cases when ruptured. Hypertension has been considered a potential risk factor for AAA; but the findings from prospective cohort studies have not been entirely consistent, nor have they been summarised in a comprehensive meta-analysis. Our aim was to conduct a systematic review and meta-analysis of cohort studies of the association between blood pressure, hypertension and AAA to clarify the strength and shape of these associations. We searched PubMed and Embase databases for relevant cohort studies up to April 30th, 2018. Random-effects models were used to calculate summary relative risks (RRs) and 95% confidence intervals (CIs). The meta-analysis included 21 cohort studies (20 publications) with data on 28,162 cases and 5,440,588 participants. The findings indicate that the RR of AAA in hypertensive patients is 1.66 times (95% CI: 1.49–1.85, I^2^ = 79.3%, *n* = 13) that of non-hypertensive patients. In addition, there was a 14% (95% CI: 6–23%, I^2^ = 30.5%, *n* = 6) and a 28% (95% CI: 12–46%, I^2^ = 80.1%, *n* = 6) increase in the RR of AAA for every 20 mmHg and 10 mmHg increase in systolic blood pressure (SBP) and diastolic blood pressure (DBP), respectively. The analysis of DBP showed evidence of a strong and highly significant nonlinear dose–response relationship (*p* < 0.001) with a steeper association from 80 mmHg and above. This meta-analysis suggests that hypertension increases the risk of developing AAA by 66%. Further studies are needed to clarify the underlying mechanism explaining the much stronger association between DBP and AAA than for SBP.

**Electronic supplementary material:**

The online version of this article (10.1007/s10654-019-00510-9) contains supplementary material, which is available to authorized users.

## Introduction

Abdominal aortic aneurysms (AAA) are expansions of the aorta at the level of the abdomen caused by the weakening of its walls [1]. Current National Institute for Health and Care Excellence (NICE) guidelines [1] define AAA as an enlargement either 1.5 times the size of the normal aorta or a diameter greater than 3 cm. Continuous stretching of the arterial walls could lead to rupture of the aorta and subsequently an internal bleeding which is fatal in roughly 80% of the cases if not treated immediately [2]. AAA’s prevalence ranges between 4 and 7% [3, 4] with more than 175,000 deaths globally attributed yearly to its rupture [5]. Most AAAs are asymptomatic and are detected either incidentally while screening for other conditions or in the event of their rupture [6]. The National Health Service (NHS) [7] exclusively offers AAA screening for men aged 65 years because the risk of AAA is considered too low in women to provide routine screening. Lederle et al. [8] have demonstrated that screening programmes that do not target high-risk populations have lower detection rates. Therefore, gathering the most evidence on the risk factors of developing AAA possibly increases the cost-effectiveness of screening programmes and detection rate of the disease.

Risk factors for the development of AAA include older age, male gender, Caucasian race, family history of the disorder, atherosclerotic disease and smoking [9, 10], the latter being considered the primary modifiable risk factor [11]. Other potential risk factors include diabetes mellitus (DM), which has been shown to be negatively associated with AAA [12], greater height [13], and low fruit and vegetable consumption [14].

Hypertension has been suggested as a risk factor for AAA [1]. It is defined by the World Health Organization (WHO) [15] as systolic blood pressure (SBP) equal to or above 140 mmHg and/or diastolic blood pressure (DBP) equal to or above 90 mmHg. The number of adults with hypertension increased from around 600 million in 1975 to 1.13 billion in 2015 [16] and has been estimated to increase to 1.5 billion by 2025 [17] due to ageing, population growth and changes in behavioural risk factors [15].

Several cross-sectional and case–control studies have examined the association between blood pressure (BP) and AAA; however, the results have not been entirely consistent with some reporting a statistically significant positive association [18–20], and others reporting no clear association [21, 22]. Prospective studies are less prone to biases and provide more reliable evidence. Some prospective studies found positive associations between BP or hypertension and AAA [5, 23–38], whereas other studies found no significant association [39–42]. There has also been considerable variation in the size of the associations reported with relative risks (RRs) varying between 1.15 and 2.19 [5, 23–42]. Previous meta-analyses on the topic have included only or mostly cross-sectional studies [43, 44], from which temporal relationships cannot be inferred. Given the mixed results of the available prospective studies, there is an urgent need to clarify the association between hypertension or BP and the risk of AAA. We therefore conducted a systematic review and meta-analysis of prospective studies on the association between hypertension or blood pressure and the risk of AAA aiming to clarify the strength and shape of the association between hypertension, BP and AAA, assess the quality of the available data and to investigate sources of heterogeneity between studies using subgroup and meta-regression analyses.

## Methods

This analysis was conducted in accordance with the preferred reporting items for systematic reviews and meta-analyses (PRISMA) guidelines. This study was registered at PROSPERO, number CRD42018098490.

### Search strategy

The PubMed and Embase databases were searched from inception up to April 30th, 2018. Identified records were first screened based on titles and abstracts then based on full texts. A manual search of secondary sources was also performed to identify articles that were missed by electronic search.

### Selection criteria

Eligibility criteria for inclusion were: (1) retrospective or prospective cohort studies, nested-case–control studies within cohort studies, or studies with follow-up periods in adult populations; (2) human-based studies; (3) available measures of association (including RRs, odds ratios and hazard ratios) adjusted for at least one confounding factor to be included in the meta-analysis of hypertension; and (4) at least three categories of SBP and DBP or a risk estimate on continuous scale had to be available for the study to be included in the dose–response analyses of SBP or DBP.

### Data extraction

Study characteristics and results were extracted into tables by one author (EK) and checked for accuracy by a second author (HP), and included the following data: first author, publication year, study location, study name or description, follow-up period, sample size with sex and age of participants, number of cases and type of outcome, exposure and subgroups, exposure categories or comparison, measure of association with a 95% confidence interval (CI) and variables adjusted for in the analysis.

### Statistical analysis

Random effects models that take into account heterogeneity between studies were used to calculate summary RR and 95% CI for the association between hypertension or BP and risk of AAA [45]. Cochrane’s Q test was used to assess the degree of heterogeneity between studies and the I^2^ statistic was reported to express the percentage of total variation across studies [46]. I^2^ values of approximately 25, 50 and 75% were considered to indicate low, moderate and high heterogeneity, respectively.

For the linear dose–response analysis, all increments for SBP and DBP were converted to 20 mmHg and 10 mmHg respectively before inclusion in the meta-analysis. When a study reported estimates for three or more categories the Greenland and Longnecker [47] method was used to calculate linear trends based on the log RR across BP categories. We used the mean or median for each category when reported by the original article, otherwise we estimated the midpoint by calculating the average of the bottom and top ranges. The lowest and highest categories were almost always open-ended; in which case we estimated open-ended interval using the same width as in the adjacent interval. Nonlinear dose–response analyses were conducted using fractional polynomial models and we determined the best fitting second order fractional polynomial regression model [48]. A likelihood ratio test was used to test for nonlinearity by comparing the nonlinear to the linear model [48].

Egger’s [49] and Begg’s [50] tests and inspection of the funnel plots were used to explore potential publication bias. When the tests indicated evidence of bias, the “Trim and Fill” method was used as a sensitivity analysis to estimate the impact of potential publication bias on the summary estimates in the meta-analysis [51]. To ensure that the results were not driven by a very large study or a study with an outlying result, sensitivity analyses were carried by omitting one study at a time from the analyses and assessing its influence on the overall summary estimate. To explore potential heterogeneity, we conducted subgroup analyses by sex, follow-up period, the definition of hypertension and its diagnostic technique, AAA diagnostic technique, geographic location, number of cases, study quality, and adjustment for a range of potentially confounding factors including race, education, height, body mass index (BMI)/weight, physical activity, smoking, alcohol intake, dyslipidaemia, hormone replacement therapy (HRT) (in women only), peripheral artery disease (PAD), cardiovascular diseases (CVDs), stroke, DM, chronic obstructive pulmonary disease (COPD), and glomerular filtration rate (GFR)/renal disease. All studies adjusted for age and therefore a subgroup analysis stratified by adjustment for age was not useful. We also inspected the forest plots to see whether there were obvious outliers that had likely substantially contributed to the observed heterogeneity. Study quality was evaluated by one author (EK) using the Newcastle–Ottawa scale (NOS) [52], a tool to assess the quality of non-randomised studies, then checked for accuracy by a second author (MH). We split the scale into three categories with scores of 0–3, 4–6 and 7–9 representing low, medium and high-quality studies respectively. All statistical tests were performed using Stata version 13.1 (StataCorp LP, College Station, TX, USA).

## Results

**Fig. 1:**
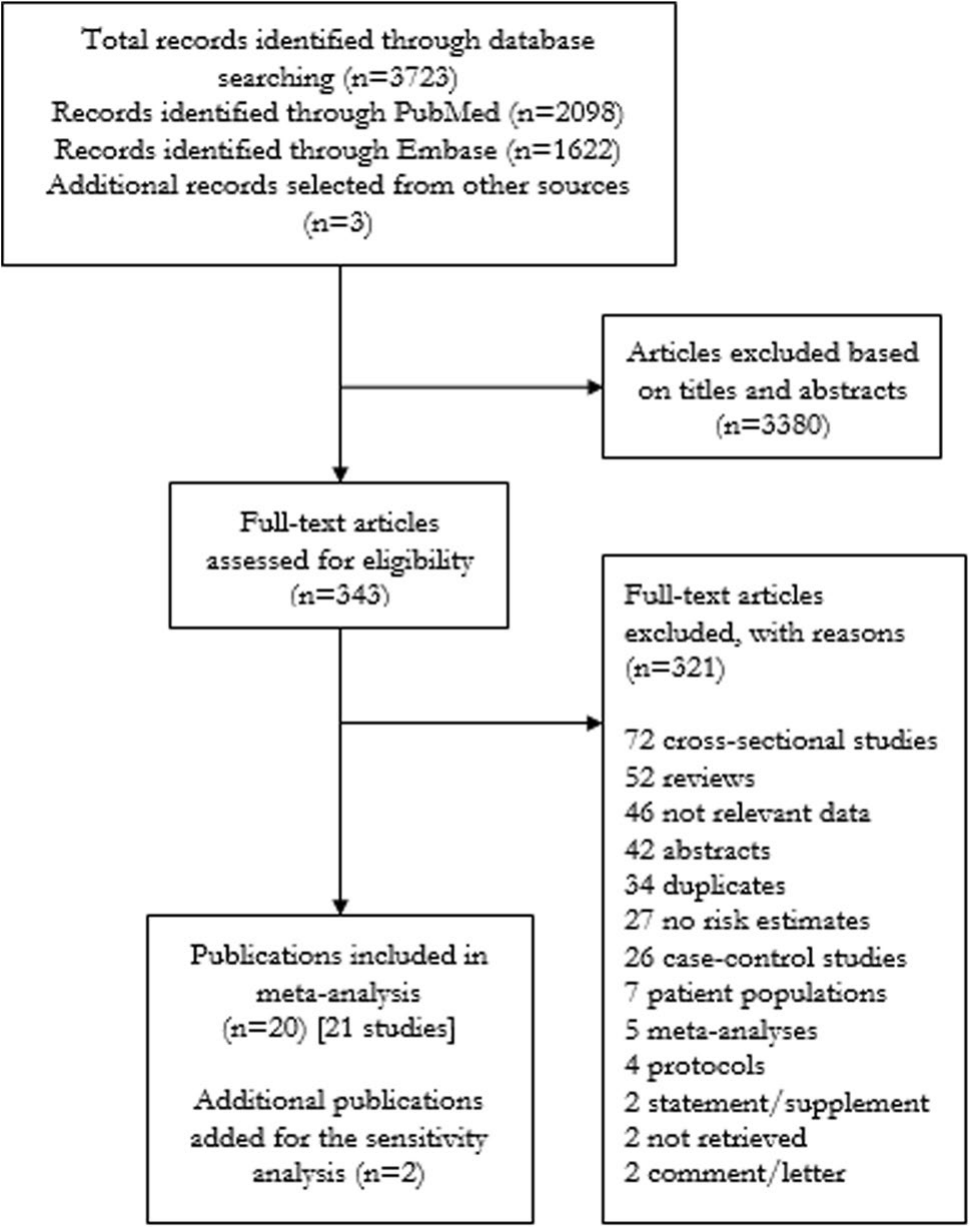
Study flow diagram. Made in accordance with PRISMA statement with modifications

A total of 3723 articles were identified, out of which 3380 articles were excluded based on their titles and abstracts. A total of 18 eligible cohort studies from 17 publications and three further articles from other sources were included in the main analyses [5, 23–42] (Fig. 1). The 21 eligible studies included data on 28,162 aortic aneurysm patients and 5,440,588 participants in total (Supplementary Tables 1a, 1b). The mean (median) NOS score was 7.4 (7), hence most of the studies were of high quality.

### Hypertension and AAA

**Fig. 2:**
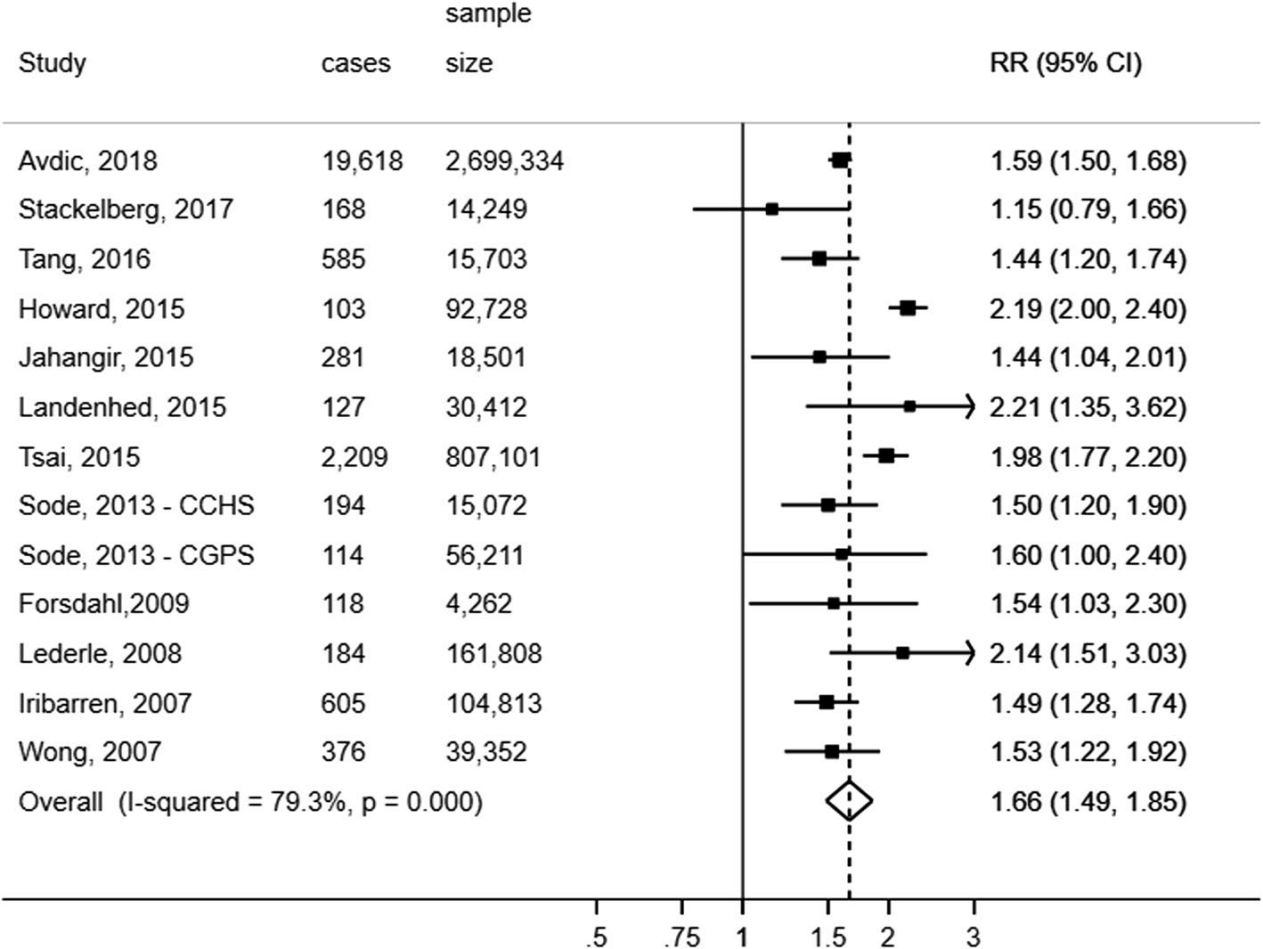
Hypertension and abdominal aortic aneurysm

Fourteen cohort studies (13 publications) [5, 28–38, 42] with a total of 26,943 cases and 5,317,552 participants were included in the meta-analysis of hypertension and risk of AAA. The summary RR for patients with hypertension versus patients without hypertension was 1.58 (95% CI: 1.32–1.90) (Supplementary Fig. 1). However, there was evidence of extreme heterogeneity (I^2^ = 96.2%, p_heterogeneity_ < 0.001), which was partly explained by a large (2261 cases and 1,258,006 participants) and outlying British study (study by Rapsomaniki et al. [33]). The lifetime AAA risk ratio comparing hypertensive and normotensive participants was 1.02 (95% CI: 0.98–1.07), a much weaker association than what was reported across the remaining studies. Consequently, we excluded the study by Rapsomaniki et al. [33] from the remaining analyses of hypertension and risk of AAA. The summary RR among the thirteen remaining cohort studies [5, 28–32, 34–38, 42] (24,682 cases, 4,059,546 participants) was 1.66 (95% CI: 1.49–1.85, I^2^ = 79.3%, p_heterogeneity_ = <0.001) (Fig. 2). The heterogeneity was further reduced when excluding the study by Howard et al. [5] (summary RR = 1.61, 95% CI: 1.47–1.76, I^2^ = 55.4%) and reduced to zero when excluding both studies by Howard et al. [5] and Tsai et al. [36] (summary RR = 1.57, 95% CI: 1.49–1.64, I^2^ = 0.0%). The summary RR ranged from 1.61 (95% CI: 1.47–1.76) when the study by Howard et al. [5] was excluded to 1.69 (95% CI: 1.52–1.89) when the study by Stackelberg et al. [42] was excluded (Supplementary Fig. 2). There was no evidence of publication bias with Egger’s test (*p* = 0.69) or Begg’s test (*p* = 0.50) and there was no evidence of asymmetry in the funnel plot (Supplementary Fig. 5).

### Systolic blood pressure and AAA

**Fig. 3:**
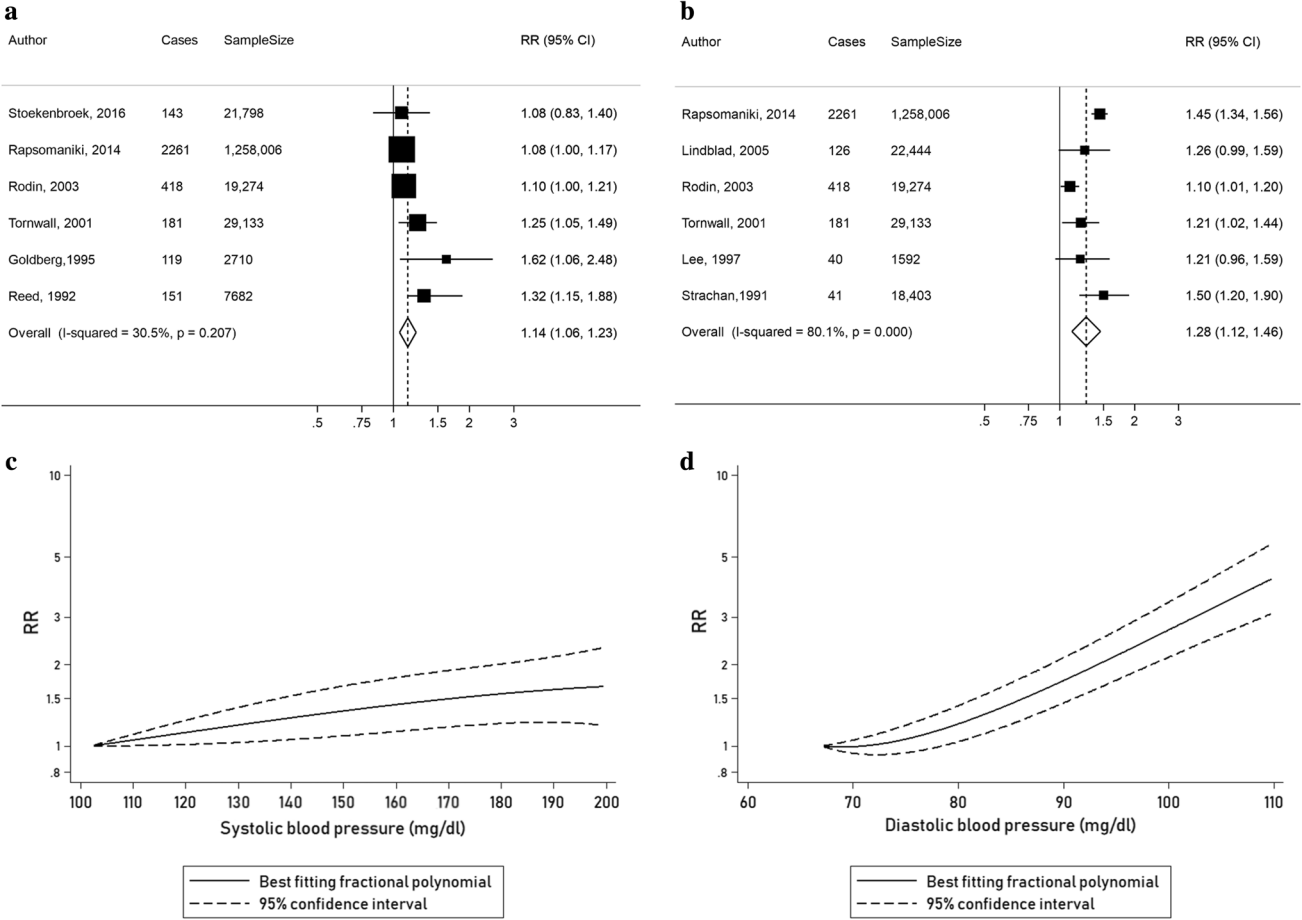
Linear and nonlinear dose–response analyses of SBP, DBP and risk of AAA, **a** linear dose–response analysis of SBP and risk of AAA, per 20 mmHg, **b** linear dose–response analysis of DBP and risk of AAA, per 10 mmHg, **c** nonlinear dose–response analysis of SBP and risk of AAA, **d** nonlinear dose–response analysis of DBP and risk of AAA

Six cohort studies [24–27, 33, 41] with a total of 3273 cases and 1,338,603 participants were included in the analysis of SBP and risk of AAA. The risk for AAA increased by 14% for every 20 mmHg increase in SBP (summary RR = 1.14, 95% CI: 1.06–1.23, I^2^ = 30.5%, p_heterogeneity_ = 0.21) (Fig. 3a). The summary RR ranged from 1.10 (95% CI: 1.05–1.18) when the study by Goldberg et al. [25] was excluded to 1.18 (95% CI: 1.05–1.33) when the study by Rodin et al. was excluded [27] (Supplementary Fig. 3). There was some evidence of publication bias in the analysis of SBP and AAA with Egger’s test (*p* = 0.045) and Begg’s test (*p* = 0.04) (Supplementary Fig. 6). Hence, the “Trim and Fill” method was used resulting in two “missing” studies being added to the analysis and an adjusted summary RR of 1.11 (95% CI: 1.02–1.22) which was not materially different from the original RR (Supplementary Fig. 7). There was a weak to moderate dose–response relationship between SBP and the risk of developing AAA (Fig. 3c); however, there was no evidence of a nonlinear relationship, p_nonlinearity_ = 0.65 (Supplementary Table 3).

### Diastolic blood pressure and AAA

Six cohort studies [23, 26, 27, 33, 39, 40] with a total of 3067 cases and 1,348,852 participants were included in the analysis of DBP and risk of AAA. The risk for developing the disease increased by 28% for every 10 mmHg increase in DBP (summary RR = 1.28, 95% CI: 1.12–1.46, I^2^ = 80.1%, p_heterogeneity_ < 0.001) (Fig. 3b). Exclusion of the study by Rodin et al. [27] reduced heterogeneity to 31.4%, while exclusion of the study by Rapsomaniki et al. [33] reduced heterogeneity to 44.4%. The summary RR ranged from  1.21 (95% CI: 1.09–1.35) when the study by Rapsomaniki et al. [33] was excluded to 1.29 (95% CI: 1.10–1.51) when the study by Tornwall et al. [26] was excluded (Supplementary Fig. 4). There was no evidence of publication bias with Egger’s test (*p* = 0.94), Begg’s test (*p* = 0.85) or by inspection of the funnel plot (Supplementary Fig. 8). There was evidence of a nonlinear and strong dose–response relationship between DBP and AAA, p_nonlinearity_ < 0.001, with a steeper increase in risk at higher levels of DBP than at lower levels (Fig. 3d, Supplementary Table 3).

### Sensitivity and subgroup analyses

Hypertension was positively associated with the risk of developing AAA in all subgroup analyses (Supplementary Table 4). There was evidence of heterogeneity when the analyses were stratified by sex (*p* for heterogeneity between subgroups = 0.01) with a summary RR of 1.46 (95% CI: 1.32–1.62, I^2^ = 0.0%) among men and 2.06 (95% CI: 1.68–2.53, I^2^ = 52.7%) among women. There was also indication of heterogeneity between subgroups when the analyses were stratified by adjustment for dyslipidaemia (*p* for heterogeneity between subgroups = 0.02), with weaker associations among studies with such adjustment (Supplementary Table 4). There was no evidence of heterogeneity in any of the remaining subgroup analyses.

## Discussion

This systematic review and meta-analysis of cohort studies comparing the risk of AAA in hypertensive versus non-hypertensive adult patients showed, overall, a 66% increased risk of developing the disease in hypertensive patients. The association between hypertension and AAA persisted in a number of subgroup and sensitivity analyses, suggesting that the results were robust.

Furthermore, a 20 mmHg increase in SBP and a 10 mmHg increase in DBP were associated with a 14% and 28% increase in the risk of developing AAA, respectively. There was evidence of a nonlinear association between DBP and AAA with a stronger increase in risk at higher levels of DBP than at lower levels, but there was no evidence of nonlinearity for SBP. Hence, the higher increase in the risk of AAA in patients with high DBP (summary RR of 6.46 for a DBP of 120 vs. 67 mmHg compared to summary RR of 1.56 for a SBP of 180 vs. 102 mmHg) suggests that DBP has a larger impact on the risk of AAA than SBP. The current findings are consistent with a pooled analysis, by the Prospective Studies Collaboration [53], in finding a positive association between SBP and risk of aortic aneurysm; however, the association was considerably stronger than in the current meta-analysis. High SBP and low DBP are associated with arterial stiffness [54], which according to Rapsomaniki et al. is protective against AAA and might explain the weaker association observed between SBP and AAA [33].

Our findings are partly consistent with a previous meta-analysis of nine cross-sectional studies [43] in finding a positive association between hypertension and AAA. However, the strength of the association was considerably stronger in the current meta-analysis of cohort studies with a 66% versus a 33% increase in the risk, respectively. The current results are likely more reliable because prospective cohort studies can better assess the temporal relationship between hypertension and AAA. In addition, the current meta-analysis included 24,682 cases and 4,059,546 participants in the analysis of hypertension and AAA (versus 1614 cases and 27,382 participants in the previous meta-analysis) and therefore had 15 times the number of cases and 148 times the number of participants compared to the previous meta-analysis, thus providing robust and precise estimates of AAA risk in hypertension.

Although the prevalence of AAA is five-fold higher in men than in women [55], our findings show that the RR of developing AAA among hypertensive women compared to non-hypertensive women was higher than the RR in hypertensive men compared to non-hypertensive men. A possible explanation is a higher prevalence of other risk factors in men, such as smoking [56], which would increase the absolute risk among men, and any additional adverse relative effect of hypertension might therefore be less than among women because of a cleaner reference group in women. The higher relative risk of AAA in women might also be explained by their higher risk of rupture and subsequently death. The UK Small Aneurysm Trial [57] showed a threefold higher risk of AAA rupture rate in women compared to men. According to Ulug et al. [58], a smaller proportion of women were eligible to undergo endovascular aneurysm repair than men and therefore a smaller proportion were offered an intervention.

Several potential limitations of this meta-analysis must be considered. There was high heterogeneity between the studies in the analyses of hypertension and DBP and the risk of AAA. However, the heterogeneity appeared to be driven more by differences in the size of the association rather than differences in the direction of the association. Subgroup analyses were conducted to investigate potential sources of heterogeneity and when studies were stratified by sex, heterogeneity substantially decreased. In addition, in the analysis of hypertension and DBP and AAA a few outlying studies [27, 33] appeared to explain a large proportion of the heterogeneity, and when excluded the heterogeneity was reduced to 0–31% while the summary estimates were not substantially altered.

The association between hypertension and AAA could potentially be confounded by other risk factors because hypertension is more common among persons with overweight and obesity, less physical activity, who smoke and who have unhealthy diets. However, the association between hypertension and AAA persisted in a number of subgroup analyses when stratified by adjustment for a range of confounding factors including race, education, height, BMI/weight, physical activity, smoking, alcohol, dyslipidaemia, HRT, PAD, CVD, stroke, DM, COPD, GFR/renal disease and there was little evidence of heterogeneity between most of these subgroups with meta-regression analyses.

Although there was some evidence of small study bias in the analyses of hypertension and SBP, the results were not substantially altered when using the “Trim and Fill” method, suggesting that small study bias is not likely to have had a large impact on the results.

Misclassification of the exposure because of self-report of hypertension diagnosis could have influenced the results; however, 10 of the 13 studies used measured blood pressure and/or medical records to assess hypertension and only 3 studies were based on self-report, thus any misclassification would most likely have had a limited impact on the summary estimates. If anything, there was a somewhat stronger association between hypertension and AAA among studies where hypertension was assessed using measured blood pressure or medical records (summary RR = 1.73, 95% CI: 1.53–1.96) than among studies with self-reported hypertension (summary RR = 1.42, 95% CI: 1.20–1.68), although the test for heterogeneity between subgroups was not significant (*p* = 0.13), thus any such misclassification would most likely be non-differential and bias the results toward the null. The studies did not take into account changes in BP or BP lowering treatment during follow-up and it is possible that a single baseline measurement may not have adequately represented long-term BP. Because of the prospective design of the included studies, any such regression dilution bias would most likely have lead to bias toward the null. However, antihypertensive medication use might have attenuated the results as it decreases BP and subsequently the effect the latter has on the risk of AAA. With regard to AAA, many cases are asymptomatic and the outcome was not always assessed by an ultrasound, but rather through medical records. Therefore, the number of cases might have been higher if diagnostic tests were performed on all participants during follow-up. However, again because of the prospective design of the included studies any such misclassification would most likely have been non-differential and led to bias towards the null.

Strengths of the current meta-analysis include the prospective design of the included studies which avoids recall bias and reduces the potential for selection bias that can affect case–control studies, and which avoids the temporal bias that may affect cross-sectional studies. The high study quality of the included studies and the large sample size (> 24,000 cases, 4 million participants for hypertension and > 3000 cases and 1.3 million participants for BP) provided sufficient statistical power to detect even moderate associations with relatively high precision. Detailed dose–response analyses clarified the strength and shape of the dose–response relationship between increasing SBP, DBP and AAA; and detailed subgroup and sensitivity analyses allowed for in-depth investigation and identification of sources of heterogeneity across studies, potential confounding as well as assessment of the robustness of the results.

The NHS exclusively offers screening to men aged 65 years due to previous evidence indicating very low risk of AAA in women [59, 60]. However, DeRubertis et al. deduced that the risk of AAA is higher in women with specific risk factors [55]. Forbes et al. [61] have shown that the threshold for diagnosing AAA is supposed to be lower in females than in males due to their aorta’s smaller size. Therefore, the prevalence of AAA in women could be underestimated in the screening studies that define aneurysms as an aortic diameter of 3 cm or higher. Our meta-analysis suggests that hypertension is a leading risk factor in women increasing their susceptibility to AAA; therefore, further prospective cohort studies in women that define AAA as a 50% increase in abdominal aortic diameter are needed to assess the relationship between hypertension, BP and risk of AAA in women.

The current meta-analysis provides the first meta-analytic evidence based only on prospective studies that hypertension and elevated BP (in particular DBP) increase the risk of AAAs. Because of the high mortality in ruptured AAAs, primary prevention may be a promising way to reduce the public health burden of this disease. The public health importance of the current analysis is illustrated by the observation that risk of AAA increased even below the cut-off point for hypertension (DBP < 90 mmHg) and there was a 75% increase in RR at around 90 mmHg compared to the reference category of 67 mmHg (Fig. 3d), suggesting that the optimal BP level may be lower than what is currently used as the cut-off for hypertension. The risk of AAA significantly increased at a DBP of 79 mmHg which is supported by pooled analyses of DBP and CVD suggesting an optimal DBP around 70–80 mmHg [53, 62, 63]. Because hypertension and elevated BP to a large degree are caused by unhealthy lifestyles, further studies are needed to clarify whether these risk factors may be directly related to AAAs. Several recent studies [42, 64, 65] from the Cohort of Swedish Men have been published in 2017 reporting strong associations between modifiable lifestyle factors such as smoking, high BMI, hypercholesterolaemia, low physical activity, and abdominal adiposity with the risk of AAA. Moreover, high intake of fruits and vegetables was associated with a decreased risk of AAA [14, 65]. It is possible therefore that elevated BP may be a mediator of the adverse effects of unhealthy lifestyle factors on AAA, but this needs further examination through additional studies as the evidence on most of these risk factors and AAA is limited.

## Conclusion

In summary, this meta-analysis found a 66% higher risk of AAA in hypertensive patients compared to non-hypertensive patients and a 14% and 28% increase in the risk of the disease for every 20 and 10 mmHg rise in SBP and DBP, respectively. Risk of AAA increased dose-dependently even within the normal BP range and there was a five to six-fold increase in the RR of AAA at the highest level of DBP compared to a 1.6 to 1.7-fold increase in the RR at the highest level of SBP based on the results from the nonlinear dose–response analysis. The mechanism underlying the much stronger association between DBP and AAA than for SBP needs further study.

## Electronic supplementary material

Below is the link to the electronic supplementary material.

Supplementary material 1 (DOCX 338 kb)
