# Supplementary material for: Blood pressure, hypertension and the risk of abdominal aortic aneurysms: a systematic review and meta-analysis of cohort studies
Source: Eur J Epidemiol. 2019 Mar 22;34(6):547–55. doi: 10.1007/s10654-019-00510-9 (PMC6497813; doi:10.1007/s10654-019-00510-9)

**Supplementary material**

**Supplementary Table 1.**

| Table 1a. Characteristics of individual cohort studies on hypertension and risk of abdominal aortic aneurysm | | | | | | | |
| --- | --- | --- | --- | --- | --- | --- | --- |
| First author, publication year, study location | **Study name or description** | **Study period** | **Number of participants, number of cases** | **Exposure and subgroup** | **Blood pressure categories, subgroup** | **Relative risk (95% confidence interval)** | **Adjustment for confounders** |
| Iribarren C et al, 2007, USA | The Kaiser Multiphasic Health Checkup Cohort Study. | 1971-2003,  13 years of follow up | 104,813 men and women, age ≥18 years  605 AAA cases | Hypertension, overall  Hypertension, men  Hypertension, women | No hypertension vs treated hypertension vs untreated hypertension  No hypertension vs treated hypertension vs untreated hypertension  No hypertension vs treated hypertension vs untreated hypertension | 1.00  2.10 (1.57-2.82)  1.31 (1.09-1.56)  1.00  2.08 (1.49-2.91)  1.26 (1.03-1.53)  1.00  2.22 (1.20-4.12)  1.59 (1.04-2.42) | Gender, age, race, education level, cigarette smoking status, alcohol consumption, height, weight, sagittal abdominal diameter, serum total cholesterol, WBC count, estimated GFR, HRT (in women), and history of CHD, DM, COPD, stroke and IC. |
| Wong D et al, 2007, USA | Health Professionals Follow-up Study. | 1986-2002,  14.6 years of follow up | 39,352 men aged 40 to 75 years.  376 AAA cases | Hypertension | Yes or No | 1.53 (1.22-1.92) | Age, smoking, DM, hypercholesterolemia, BMI, physical activity. |
| Lederle F et al, 2008, USA | The women’s health  Initiative. | 1993/8 - 2004/5  7.8 years of follow up | 161,808 women aged 50-79 years.  184 AAA cases. | Hypertension | Yes vs No | 2.14 (1.51-3.03) | Participation in clinical trial, assigned treatment, age, race, height, weight, smoking status, pack years, drugs for hypercholesterolemia, CAD, CVD, PAD, VTE, DM, COPD, non-skin cancer, history of AAA, history and current hormone therapy and alcohol use. |
| Forsdahl SH et al, 2009, Norway | The Tromsø Study | 1994–2001,  7 years of follow up | 4262 men and women, ages between 25 and 82 years  118 AAA cases | Hypertension | Yes vs No | 1.54 (1.03-2.30) | Age, sex, serum total cholesterol, serum HDL, use of statins, smoking |
| Sode BF et al, 2013, Denmark | Copenhagen City Heart Study | 1976-2003,  23 years of follow up | 15,072 men and women, age ≥20 years  335 AA cases | Hypertension | Yes vs No | 1.5 (1.2-1.9) | Age. smoking, sex. Hypercholesterolemia, BMI, alcohol, DM |
| Sode BF et al, 2013, Denmark | Copenhagen General Population Study | 2003-2010,  4 years of follow up | 56,211 men and women, age ≥20 years  169 AA cases. | Hypertension | Yes vs No | 1.6 (1.0-2.4) | Age. smoking, sex. Hypercholesterolemia, BMI, alcohol, DM |
| Rapsomaniki E et al, 2014, UK | CArdiovascular research using LInked Bespoke studies and Electronic health Records programme | 1997-2010,  5.2 years of follow up | 1,258,006 men and women, age ≥30 years  2261 AAA cases | Hypertension | Yes vs No | 1.02 (0.98-1.07) | Age, sex and primary care practice |
| Howard DPJ et al, 2015, UK | The Oxford Vascular Study (OXVASC) | 2002-2014,  12 years of follow up | 92,728 men and women above the age of 55.  103 AAA cases | Hypertension, men  Hypertension, women | Yes vs No  Yes vs No | 1.60(1.32-1.94)  2.41(2.17-2.68) | Age and sex |
| Jahangir E et al, 2015, USA | Southern Community Cohort Study | 2002-2012,  4.98 years of follow up | 18,501 men and women, age ≥65 years  281 AAA cases | History of high BP, all  History of high BP, men  History of high BP, women | Yes vs. No  Yes vs. No  Yes vs. No | 1.44 (1.04-2.01)  1.36 (0.90-2.06)  1.57 (0.90-2.06) | Age, sex, race, BMI, education and history of smoking, MI/CABG, cholesterol, and DM. |
| Landenhed M et al, 2015, Sweden | Malmö Diet and Cancer Study | 1991-2011  16 years of follow-up | 30,412 men and women, average age 58 years  127 AAA cases | Hypertension | Yes vs No | 2.21 (1.35-3.62) | Age, sex, smoking, diabetes, obesity, apoA1, apoB |
| Tsai CL et al, 2015, Taiwan | The National Health Research Institute | 1998-2010  12 years of follow up | 807,101 men and women aged 54 and above  2209 AA cases | Hypertension | Yes vs No | 1.98(1.77-2.20) | Age, sex, urbanization, occupation, monthly income, T2DM, CKD, IHD, stroke, retinopathy, blindness |
| Tang W et al, 2016, USA | The ARIC study | 1987 – 2011,  22.5 years of follow up | 15,703 men and women, age 45-64 years  585 AAA cases | Hypertension | Yes vs No | 1.44 (1.20-1.74) | Age, sex, race, height, pack-years of smoking, alcohol consumption, HDL-C, LDL-C, TG, PAD and DM. |
| Stackelberg O et al, 2017, Sweden | Population-based Cohort of Swedish Men | 1998-2015,  13 years of follow up | 14,249 men aged 45 years and older.  168 AAA cases. | Hypertension | Yes vs No | 1.15(0.79-1.66) | Education, smoking, BMI, waist, walking/bicycling, healthy diet score, alcohol, DM, CVD, hypercholesterolemia, total energy intake |
| Avdic T et al, 2018, Sweden | Swedish National Diabetes Register | 1998-2015  7 years of follow up | 2,699,334 men and women aged ≥ 18 years.  19,618 AA cases | Hypertension | Yes vs No | 1.59 (1.50–1.68) | Age, sex, DM complication, stroke, CVD, CHD, MI, AFib, renal dysfunction, psychiatric disorders, dementia, cancer, gastric bypass, medications, country of birth, marital status, education level |
| AFib = atrial fibrillation; BMI = body mass index;CABG =coronary artery bypass graft; CAD = coronary artery disease;CHD = coronary heart disease; CKD = chronic kidney disease; COPD = chronic obstructive pulmonary disease; DM = diabetes mellitus; FEV = Forced Expiratory Volume; GFR = glomerular filtration rate; HDL = high-density lipoprotein; HRT = hormone replacement therapy; IC = intermittent claudication; IHD =ischemic heart disease; LDL = low-density lipoprotein; MI = myocardial infarction; PAD = peripheral arterial disease; TG = triglycerides; VTE = venous thromboembolism; WBC = white blood cells. | | | | | | | |

| Table 1b. Characteristics of individual cohort studies on blood pressure and risk of abdominal aortic aneurysm | | | | | | | |
| --- | --- | --- | --- | --- | --- | --- | --- |
| First author, publication year, study location | **Study name or description** | **Study period** | **Number of participants, number of cases** | **Exposure and subgroup** | **Blood pressure categories, subgroup** | **Relative risk (95% confidence interval)** | **Adjustment for confounders** |
| Strachan DP et al, 1991, UK | The Whitehall study | 1967/9-1987  16.6 years of follow-up | 18,403 men aged 40-64 years  41 AAA cases | Diastolic blood pressure | Per 10mmHg | 1.5 (1.2-1.9) | Smoking |
| Reed D et al, 1992, Hawaii | Honolulu Heart Program | 1965/8-1988  20 years of follow-up | 7682 men aged ≥ 46 years  151 AA cases | Systolic blood pressure | Per 50mmHg | 2.01 (1.41-4.84) | Age, serum cholesterol and triglyceride, cigarette pack-years, height |
| Goldberg RJ, 1995, Hawaii | Honolulu Heart Program | 1965/8-1988  20 years of follow-up | 2710 men between the ages of 55-64  119 AA cases | Systolic blood pressure | ≤120.7 mmHg  121.3-134.3  134.7-149.3  ≥150 | 1.00  1.56 (0.53-4.62)  3.38 (1.23-9.26)  2.96 (1.06-8.28) | Age, ventricular rate, BMI, serum cholesterol, triglyceride, glucose and uric acid, hematocrit, FEV, physical activity, smoking, alcohol |
| Lee AJ et al, 1997, UK | The Edinburgh Artery Study | 1987/8-1992/4, 5 years of follow up | 1592 men and women aged 55 to 74 years.  40 AAA cases. | Diastolic blood pressure | Per 5mmHg | 1.10 (0.98-1.26) | Age, sex and atherosclerotic disease. |
| Tornwall ME et al, 2001, Finland | AAA within ATBC Study | 1985/8-1993,  5.8 years of follow up | 29,133 men aged 50 to 69 years.  181 AAA cases | Systolic blood pressure  Diastolic blood pressure | ≤130 mmHg  131-160  >160  ≤85 mmHg  86-100  >100 | 1.00  1.34 (0.87-2.07)  1.95 (1.15-3.30)  1.00  1.35 (0.93-1.94)  1.78 (1.03-3.05) | Age, cigarettes/day, years of smoking, BMI, total cholesterol, HDL, history of DM, education, exercise, alpha-tocopherol and beta-carotene supplementation group |
| Rodin M et al, 2003, USA | Chicago Heart Association Detection Project in Industry cohort | 1967/73-2000, 30 years of follow up | 19,274 men and women 40 to 64 years of age.  418 AAA cases. | Systolic blood pressure  Diastolic blood pressure | Per 20mmHg  Per 12mmHg | 1.10 (coeff = 0.0049;  t = 1.99)  1.12 (coeff = 0.0094;  t = 2.18) | Serum cholesterol, ex vs never smoker, cigarettes/day, height, gender and age. |
| Lindblad B et al, 2005, Sweden | Malmo¨ Prevention Project | 1974–1991,  21 years of follow up | 22,444 men  126 AAA cases | Diastolic blood pressure | Per 11mmHg | 1.29 (0.99-1.67) | Age, S-TG, S-cholesterol, physical inactivity, smoking |
| Rapsomaniki E et al, 2014, UK | CArdiovascular research using LInked Bespoke studies and Electronic health Records programme | 1997-2010,  5.2 years of follow up | 1,258,006 men and women, age ≥30 years  2261 AAA cases | Systolic blood pressure  Diastolic blood pressure  Systolic blood pressure, age 30-59 years  Systolic blood pressure, age 60-79 years  Systolic blood pressure, age ≥80 years  Diastolic blood pressure, age 30-59 years  Diastolic blood pressure, age 60-79 years  Diastolic blood pressure, age ≥80 years | Per 20mmHg  Per 10mmHg  90-114 mmHg  115-129  130-139  140-159  160-179  ≥180  90-114 mmHg  115-129  130-139  140-159  160-179  ≥180  90-114 mmHg  115-129  130-139  140-159  160-179  ≥180  60-74 mmHg  75-84 mmHg  85-89  90-94  95-99  ≥100  60-74 mmHg  75-84  85-89  90-94  95-99  ≥100  60-74 mmHg  75-84  85-89  90-94  95-99  ≥100 | 1.08 (1.00-1.17)  1.45 (1.34-1.56)  0.93 (0.51-1.67)  1.05 (0.86-1.15)  1.25 (0.84-1.87)  1.70 (1.10-2.62)  2.60 (1.45-4.65)  3.97 (1.74-9.04)  0.95 (0.80-1.14)  1.02 (0.95-1.10)  1.07 (0.85-1.34)  1.10 (0.81-1.51)  1.12 (0.82-1.52)  1.12 (0.77-1.65)  0.88 (0.62-1.26)  1.05 (0.91-1.23)  1.17 (0.74-1.85)  1.27 (0.65-2.49)  1.25 (0.64-2.44)  1.19 (0.57-2.48)   - 1. (0.63-1.62)   1.06 (0.96-1.18)  1.42 (1.15-1.76)  1.82 (1.34-2.48)  2.34 (1.53-3.58)  3.40 (1.83-6.33)  0.88 (0.70-1.11)  1.07 (1.00-1.15)  1.37 (1.20-1.56)  1.71 (1.46-2.00)  2.15 (1.74-2.66)  3.04 (2.21-4.19)  0.66 (0.40-1.08)  1.18 (1.04-1.33)  1.68 (1.35-2.09)  2.14 (1.60-2.85)  2.72 (1.84-4.01)  3.89 (2.22-6.83) | Age, sex and primary care practice |
| Stoekenbroek R et al, 2016, UK | The EPIC-Norfolk prospective population study | 1993/7 – 2008, 12.1 years of follow up | 21,798 men and women aged between 45 and 79 years old  143 AAA cases | Systolic blood pressure | <122 mmHg  122-133  134-146  >146 | 1.00  1.45 (0.78-2.70)  1.29 (0.69-2.38)  1.30 (0.70-2.39) | Age, sex, smoking, BMI, DM, HDL-C and LDL-C |
| BMI = body mass index; DM = diabetes mellitus; FEV =Forced Expiratory Volume; HDL = high-density lipoprotein; LDL = low-density lipoprotein; TG = triglycerides. | | | | | | | |

**Supplementary Table2. Definitions and diagnostics of hypertension and AAA**

| Author | | | | HTN | | | | | AAA | | | |
| --- | --- | --- | --- | --- | --- | --- | --- | --- | --- | --- | --- | --- |
| Year | | | **Definition** | | **Diagnosis** | | | | **Definition** | | | **Diagnosis** |
| Strachan 1991 | | | continuous | | measured | | | | death attributed to AAA | | | death certificates |
| Reed, 1992 | | | continuous | | average of 3 measurements | | | |  | | | based on all available medical, surgical, and autopsy records |
| Goldberg, 1995 | | | systolic (≤121.2, 121.3–134.3, 134.3-149.3 and >160 mmHg) | | average of 3 measurements | | | |  | | | based on all available medical, surgical, and autopsy records |
| Lee, 1997 | | | continuous | | measured | | | | ≥ 3cm | | | ultrasound scans |
| Tornwall, 2001 | | | systolic (≤130, 131–160, and >160 mmHg) and diastolic (≤85, 86–100, and >100 mmHg) blood pressure | | measured | | | | either ruptured or non-ruptured | | | registers, hospital and autopsy records |
| Rodin, 2003 | | | continuous | | self-administered questionnaire | | | |  | | | Death certificates + Medicare records |
| Lindblad, 2005 | | | continuous | | measured | | | | symptomatic or large (5–5.5 cm in diameter) AAA | | | based on hospital register data, SwedVasc quality control data and death certificates |
| Iribarren, 2007 | | | SBP> 140mmHg or DBP>90mmHg or treatment | | measured + self-reported physician diagnosed hypertension/antihypertensive use | | | |  | | | hospitalizations with primary discharge diagnosis of AAA |
| Wong, 2007 | |  | | | self-reported | | | > 3cm / repair / death | | | medical records | |
| Lederle, 2008 | | | >140/90 mmHg or treatment | | measured | | | | symptomatic or required intervention, or both, and had a diagnostic or interventional procedure that demonstrated the aneurysm | | | medical records |
| Forsdahl, 2009 | | | SBP> 160mmHg or DBP>95mmHg | | measured | | | | (1) The aortic diameter at the level of the renal arteries was ≥35 mm; (2) the infrarenal aortic diameter was ≥5 mm larger than the diameter at the level of renal arteries; or (3) a localized dilatation of the aorta was present on ultrasound | | | ultrasound scans |
| Sode, 2013 | | | SBP>140mmHg or DBP>90mmHg or treatment | | measured | | | | 50% increase in the diameter or a diameter ≥ 3cm | | | registry |
| Rapsomanik, 2014 | | | ≥ 140/90 or diagnosed by a physician or on treatment | | medical records in primary care | | | | ruptured or non-ruptured | | | diagnosed in primary care, secondary care, or at death |
| Howard, 2015 | | |  | | medical record at the GP | | | |  | | | searches for acute events in hospital and retrospective searches of hospital, primary care administrative and diagnostic coding data, and centralized death certification |
| Jahangir, 2015 | | |  | | self-reported | | | | 1.5 times the usual diameter or a diameter ≥ 3cm | | | Medicare (ruptured or non-ruptured) |
| Landenhed, 2015 | | | On treatment or BP≥140/90 mmHg | | Measured | | | |  | | | Swedish national registers |
| Tsai, 2015 | | |  | | inpatient records | | | |  | | | hospital discharge records |
| Stoekenbroek, 2016 | continuous | | | | |  | ruptured or non-ruptured | | | death certificates + hospital records | | |
| Tang, 2016 | | | SBP≥ 140mmHg or DBP≥90mmHg or on treatment | | average of the last 2 measurements out of 3 | | | | ≥ 3cm | | | hospitalization and death records as well as Medicare data + abdominal ultrasound (to identify asymptomatic AAA) |
| Stackelberg, 2017 | | |  | | self-reported | | | | ≥ 3cm | | | ultrasound scans |
| Avdic, 2018 | | | 3 filled prescriptions for antihypertensive medication for 1 year before index date | | Prescribed Drug Register | | | | hospitalization for AA and death | | | national databases and registries |

**Supplementary Table 3. Table of relative risks (95% confidence intervals) from the nonlinear dose-response analysis of systolic and diastolic blood pressure and risk of AAA**

| **SBP** | **RR (95% CI)** | **DBP** | **RR (95% CI)** |
| --- | --- | --- | --- |
| 102 | 1.00 | 67 | 1.00 |
| 105 | 1.02 (1.00-1.04) | 70 | 0.99 (0.94-1.05) |
| 110 | 1.05 (1.00-1.11) | 75 | 1.06 (0.95-1.20) |
| 115 | 1.09 (1.01-1.17) | 80 | 1.21 (1.04-1.41) |
| 120 | 1.12 (1.01-1.24) | 85 | 1.44 (1.21-1.72) |
| 125 | 1.16 (1.02-1.32) | 90 | 1.75 (1.45-2.13) |
| 130 | 1.20 (1.03-1.39) | 95 | 2.17 (1.75-2.68) |
| 135 | 1.23 (1.04-1.46) | 100 | 2.70 (2.13-3.41) |
| 140 | 1.27 (1.06-1.53) | 105 | 3.37 (2.59-4.38) |
| 145 | 1.31 (1.07-1.60) | 110 | 4.20 (3.12-5.66) |
| 150 | 1.35 (1.09-1.67) | 115 | 5.22 (3.73-7.31) |
| 155 | 1.39 (1.11-1.73) | 120 | 6.46 (4.42-9.43) |
| 160 | 1.42 (1.13-1.13) |  |  |
| 165 | 1.46 (1.15-1.85) |  |  |
| 170 | 1.50 (1.18-1.90) |  |  |
| 175 | 1.53 (1.20-1.96) |  |  |
| 180 | 1.56 (1.21-2.01) |  |  |
| 185 | 1.59 (1.22-2.07) |  |  |
| 190 | 1.62 (1.22-2.14) |  |  |
| 195 | 1.64 (1.21-2.22) |  |  |
| 200 | 1.66 (1.19-2.32) |  |  |
| p_nonlinearity_ | 0.65 | p_nonlinearity_ | <0.001 |

**Supplementary Table 4. Subgroup analyses of hypertension and risk of abdominal aortic aneurysm**

|  | n | Relative Risk  (95% CI) | I^2^ (%) | P_h_^1^ | P_h_^2^ |
| --- | --- | --- | --- | --- | --- |
| All studies | 13 | 1.66(1.49-1.85) | 79.3 | <0.001 |  |
| Sex  Men  Women  Men & Women | 5  4  7 | 1.46(1.32-1.62)  2.06(1.68-2.53)  1.66(1.47-1.86) | 0.0  52.7  63.9 | 0.53  0.10  0.01 | 0.01 |
| Follow-up  < 10 years  10-19 years  ≥ 20 years | 5  6  2 | 1.60(1.51-1.69)  1.74(1.46-2.07)  1.46(1.27-1.69) | 0.0  83.5  0.0 | 0.53  <0.001  0.79 | 0.64 |
| HTN diagnostic method  Self-reported  Measured/Records | 3  10 | 1.42(1.20-1.68)  1.73(1.53-1.96) | 0.0  82.5 | <0.001  0.44 | 0.13 |
| Hypertension definition  Current  Not current  Not defined | 6  2  5 | 1.57(1.40-1.76)  1.59(1.50-1.68)  1.72(1.43-2.06) | 19.9  0.0  81.3 | 0.28  0.88  <0.001 | 0.46 |
| AAA diagnostic method  Ultrasound  Other^ǂ^ | 3  10 | 1.40(1.20-1.63)  1.73(1.53-1.96) | 0.0  81.8 | 0.50  <0.001 | 0.11 |
| Geographic location  America  Europe  Asia | 5  7  1 | 1.52(1.38-1.68)  1.66(1.39-2.00)  1.98(1.78-2.21) | 4.9  85.4  - | 0.38  <0.001  - | 0.58 |
| Number of cases  <250  250-500  >500 | 7  2  4 | 1.74(1.41-2.13)  1.50(1.24-1.81)  1.63(1.43-1.86) | 71.8  0.0  81.3 | 0.002  0.77  0.001 | 0.75 |
| Study quality*  0-3 stars  4-6 stars  7-9 stars | 0  3  10 | -  1.49(1.33-1.68)  1.71(1.50-1.96) | -  0.0  82.8 | -  0.96  <0.001 | 0.32 |
| Adjustment for confounding factors | | | | | |
| Race  Yes  No | 4  8 | 1.54(1.34-1.76)  1.70(1.48-1.95) | 28.6  83.4 | 0.24  <0.001 | 0.45 |
| Education  Yes  No | 4  9 | 1.54(1.42-1.66)  1.77(1.55-2.03) | 16.4  71.6 | 0.31  <0.001 | 0.10 |
| Height  Yes  No | 4  9 | 1.50(1.27-1.79)  1.73(1.51-1.97) | 51.2  82.1 | 0.11  <0.001 | 0.23 |
| BMI/weight  Yes  No | 8  5 | 1.54(1.39-1.72)  1.76(1.48-2.09) | 15.5  90.8 | 0.31  <0.001 | 0.24 |
| Physical activity  Yes  No | 2  11 | 1.38(1.05-1.81)  1.71(1.52-1.92) | 39.6  81.0 | 0.198  <0.001 | 0.19 |
| Smoking  Yes  No | 9  4 | 1.54(1.40-1.69)  1.79(1.49-2.15) | 3.4  93.0 | 0.41  <0.001 | 0.19 |
| Alcohol  Yes  No | 6  7 | 1.50(1.34-1.68)  1.77(1.52-2.06) | 19.9  85.9 | 0.28  <0.001 | 0.12 |
| Dyslipidaemia  Yes  No | 9  4 | 1.49(1.37-1.62)  1.93(1.59-2.34) | 0.0  92.4 | 0.79  <0.001 | 0.02 |
| HRT  Yes  No | 2  11 | 1.72(1.22-2.44)  1.71(1.50-1.96) | 71.3  83.1 | 0.06  <0.001 | 0.94 |
| PAD  Yes  No | 2  11 | 1.71(1.16-2.51)  1.66(1.47-1.87) | 74.2  81.2 | 0.05  <0.001 | 0.96 |
| CVD  Yes  No | 3  10 | 1.59(1.23-2.04)  1.68(1.47-1.92) | 65.0  76.9 | 0.06  <0.001 | 0.68 |
| Stroke  Yes  No | 3  10 | 1.68(1.44-1.96)  1.64(1.39-1.94) | 85.9  75.5 | 0.001  <0.001 | 0.89 |
| Diabetes mellitus  Yes  No | 11  2 | 1.61(1.46-1.76)  1.94(1.40-2.70) | 59.3  64.5 | 0.01  0.1 | 0.08 |
| COPD  Yes  No | 2  11 | 1.72(1.22-2.44)  1.65(1.46-1.87) | 71.3  81.2 | 0.06  <0.001 | 0.87 |
| GFR/renal disease  Yes  No | 3  10 | 1.68(1.44-1.96)  1.64(1.39-1.94) | 85.9  75.5 | 0.001  <0.001 | 0.89 |
| * star given for follow-up period > 5 years & when loss to follow-up ≤ 10%  ǂ: medical records, death certificates, registries  n: number of studies  P_h_^1^: p-value for heterogeneity within each subgroup.  P_h_^2^: p-value for heterogeneity between subgroups with meta-regression analysis. | | | | | |

**Supplementary Table 5. Subgroup analyses of systolic blood pressure and risk of abdominal aortic aneurysm**

|  | n | Relative Risk  (95% CI) | I^2^ (%) | P_h_^1^ | P_h_^2^ |
| --- | --- | --- | --- | --- | --- |
| All studies | 6 | 1.14(1.06-1.23) | 30.2 | 0.209 |  |
| Sex  Men  Women  Men & Women | 3  0  3 | 1.30(1.14-1.49)  -  1.09(1.03-1.15) | 0.0  -  0.0 | 0.54  -  0.96 | 0.07 |
| Follow-up  < 10 years  10-19 years  ≥ 20 years | 2  1  3 | 1.14(0.99-1.30)  1.08(0.83-1.40)  1.24(1.02-1.51) | 55.2  -  55.8 | 0.14  -  0.10 | 0.65 |
| Geographic location  America  Europe | 3  3 | 1.11(1.02-1.21)  1.24(1.02-1.51) | 11.6  55.8 | 0.32  0.10 | 0.57 |
| Adjustment for confounding factors | | | | | |
| Height  Yes  No | 2  4 | 1.16(0.99-1.37)  1.16(1.02-1.31) | 45.6  43.4 | 0.18  0.15 | 1.00 |
| BMI/weight  Yes  No | 3  3 | 1.24(1.05-1.46)  1.10(1.03-1.18) | 22.9  13.9 | 0.27  0.31 | 0.25 |
| Smoking  Yes  No | 4  2 | 1.17(1.04-1.31)  1.15(0.96-1.39) | 31.7  57.0 | 0.22  0.13 | 0.78 |
| Total cholesterol  Yes  No | 4  2 | 1.22(1.07-1.38)  1.08(1.00-1.16) | 43.6  0.0 | 0.15  1.00 | 0.29 |
| Diabetes mellitus  Yes  No | 3  3 | 1.24(1.05-1.46)  1.10(1.03-1.18) | 22.9  13.9 | 0.27  0.31 | 0.25 |
| * star given for follow-up period > 5 years & when loss to follow-up ≤ 10%  ǂ: medical records, death certificates, registries  n: number of studies  P_h_^1^: p-value for heterogeneity within each subgroup.  P_h_^2^: p-value for heterogeneity between subgroups with meta-regression analysis. | | | | | |

**Supplementary Table 6. Subgroup analyses of diastolic blood pressure and risk of abdominal aortic aneurysm**

|  | n | Relative Risk  (95% CI) | I^2^ (%) | P_h_^1^ | P_h_^2^ |
| --- | --- | --- | --- | --- | --- |
| All studies |  |  |  |  |  |
| Sex  Men  Women  Men & Women | 3  0  3 | 1.30(1.14-1.47)  -  1.25(1.01-1.55) | 9.8  -  91.3 | 0.33  -  <0.001 | 0.74 |
| Follow-up  < 10 years  10-19 years  ≥ 20 years | 2  1  3 | 1.35(1.13-1.60)  1.50(1.19-1.89)  1.12(1.04-1.21) | 71.8  -  0.0 | 0.06  -  0.48 | 0.13 |
| Geographic location  America  Europe | 2  4 | 1.11(1.02-1.20)  1.37(1.24-1.52) | 0.0  35.6 | 0.48  0.20 | 0.06 |
| Study quality  0-3 stars  4-6 stars  7-9 stars | 0  2  4 | -  1.38(1.16-1.64)  1.24(1.05-1.47) | -  6.0  87.1 | -  0.30  <0.001 | 0.50 |
| Adjustment for confounding factors | | | | | |
| Smoking  Yes  No | 3  3 | 1.25(1.03-1.51)  1.32(1.15-1.52) | 70.6  58.7 | 0.03  0.09 | 0.63 |
| Dyslipidaemia  Yes  No | 4  2 | 1.14(1.06-1.22)  1.45(1.35-1.56) | 0.0  0.0 | 0.56  0.78 | 0.01 |
| * star given for follow-up period > 5 years & when loss to follow-up ≤ 10%  ǂ: medical records, death certificates, registries  n: number of studies  P_h_^1^: p-value for heterogeneity within each subgroup.  P_h_^2^: p-value for heterogeneity between subgroups with meta-regression analysis. | | | | | |

**Supplementary Figure 1. Risk of AAA in patients with hypertension versus patients without hypertension (including CALIBER study)**

**Supplementary Figure 2. Influence of each study on the overall meta-analysis summary estimate of hypertension and risk of AAA**

**Supplementary Figure 3. Influence of each study on the overall meta-analysis summary estimate of systolic blood pressure and risk of AAA**

**Supplementary Figure 4. Influence of each study on the overall meta-analysis summary estimate of diastolic blood pressure and risk of AAA**

**Supplementary Figure 5. Funnel plot of hypertension and risk of abdominal aortic aneurysm**


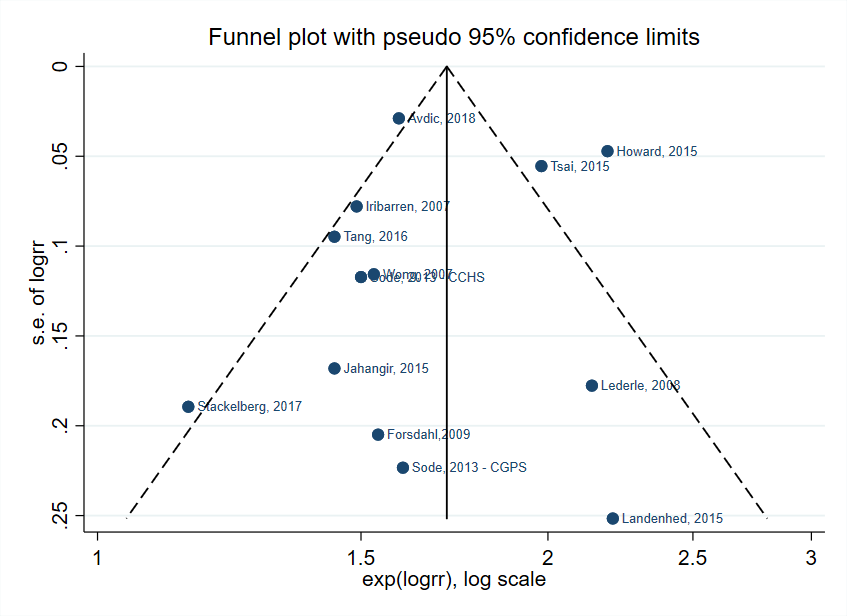


**Supplementary Figure 6. Funnel plot of systolic blood pressure and risk of abdominal aortic aneurysm**


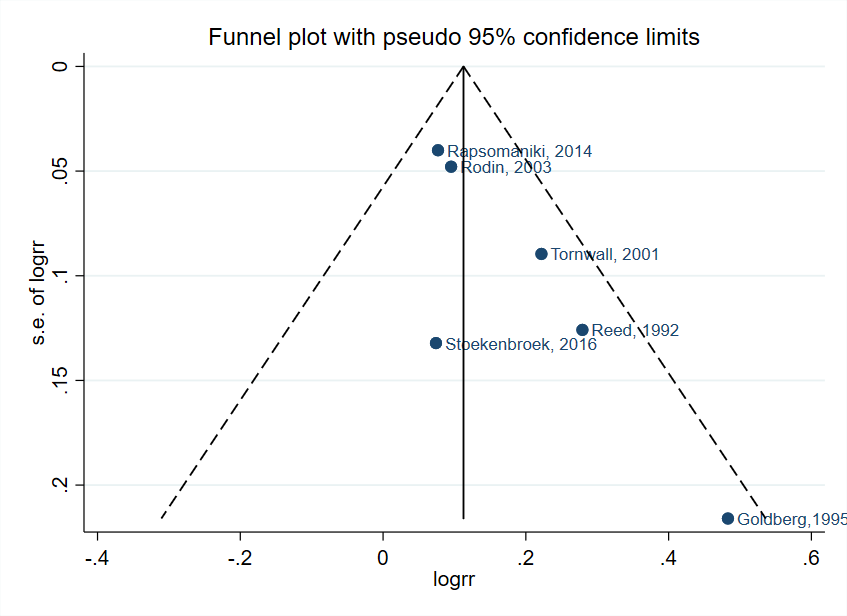


**Supplementary Figure 7.“Trim and Fill” plot for systolic blood pressure and abdominal aortic aneurysm**

**Supplementary Figure 8. Funnel plot for diastolic blood pressure and risk of abdominal aortic aneurysm**


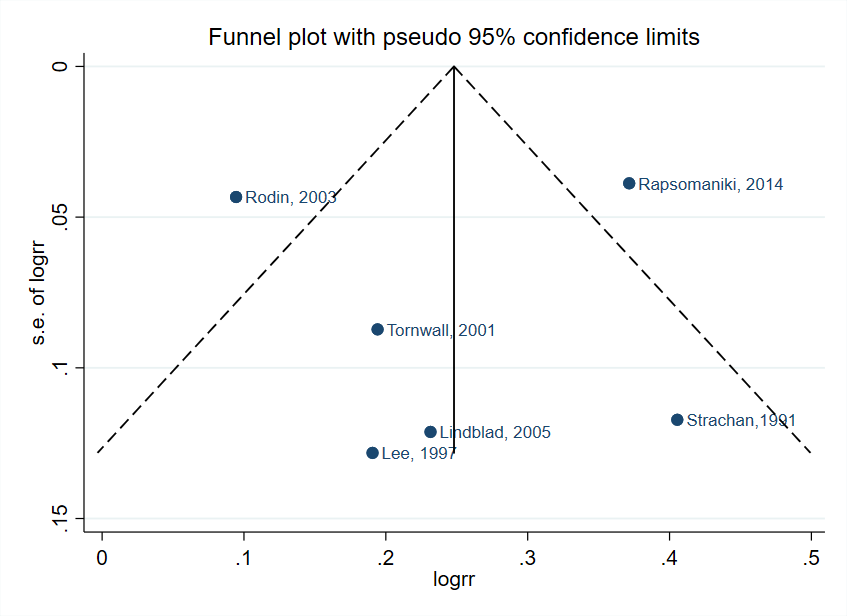

Supplement: Supplementary file 1 — Supplementary material 1 (DOCX 338 kb) [file 10654_2019_510_MOESM1_ESM.docx]
